# Analysis of preoperative and postoperative depression and anxiety in patients with osteochondral lesions of the talus

**DOI:** 10.3389/fpsyt.2024.1356856

**Published:** 2024-03-08

**Authors:** ShiHang Cao, Qiang Zan, Jun Lu, Yi Li, BingBing Li, Hang Zhao, Tao Wang, JunKui Xu

**Affiliations:** ^1^ Honghui Hospital, Xi’an Jiaotong University, Xi’an, Shaanxi, China; ^2^ Department of Joint Surgery, Affiliated Hospital of Shaanxi University of Traditional Chinese Medicine, Xianyang, Shaanxi, China; ^3^ Department of Joint Surgery, Huaibei Miners General Hospital, Huaibei, Anhui, China

**Keywords:** osteochondral lesions of the talus, autologous osteoperiosteal transplantation, depression, anxiety, prognosis

## Abstract

**Purpose:**

This study aims to investigate the psychological status of patients with Hepple V osteochondral lesions of the talus (OLT) and evaluate the effect of autologous osteoperiosteal transplantation (AOPT) on their psychological well-being.

**Methods:**

Fifty patients with Hepple V OLT who underwent AOPT at the Comprehensive Foot and Ankle Surgery Ward of Xi’an Honghui Hospital from November 2021 to May 2023 were included in this study. The patients were divided into two groups based on the presence or absence of preoperative symptoms of anxiety/depression. Group A comprised patients with preoperative symptoms, while Group B included patients without such symptoms. Preoperative and final follow-up assessments included the Hospital Anxiety and Depression Scale for evaluating anxiety and depression, the visual analogue scale for pain assessment, and the American Orthopaedic Foot and Ankle Society scores for assessing ankle and hindfoot function.

**Results:**

Among the 50 Hepple V OLT patients who obtained complete follow-up, twenty-four had preoperative symptoms of anxiety/depression, with an incidence rate of up to 48%. Patients in Groups A and B showed significant improvement in all evaluation indexes after AOPT compared to the preoperative period, but the overall prognosis of Group A was poorer than that of Group B.

**Conclusion:**

AOPT can effectively improve patients’ pain, functional activities, and psychological status, and there is a significant correlation between patients’ preoperative psychological status and prognosis.

## Introduction

Osteochondral Lesions of the Talus (OLT) is an injury to the cartilage of the talus dome with involvement of the subchondral bone, which usually occurs after ankle injuries (1). OLT as a chronic progressive disease, is often the main cause of chronic pain in the ankle joint. It is characterized by swelling and pain in the affected ankle joint, and in severe cases, it can lead to ankle stiffness and limited mobility (2). For patients with Hepple V OLT, conservative treatment cannot achieve good results, and the persistent symptoms may have a huge psychological burden on the patients. Currently, Autologous osteoperiosteal transplantation (AOPT) has been shown to be a safe and effective treatment for patients with Hepple V OLT, as it can relieve pain, improve functional ankle motion and quality of life (3–5).

The study found that patients with preoperative anxiety and depression had poorer outcomes and satisfaction after total knee arthroplasty and total hip arthroplasty (6–8). Furthermore, there is a certain correlation between psychological state and patient symptoms, with patients experiencing poorer psychological state being more sensitive to symptoms (9, 10). Therefore, optimizing the preoperative psychological state of patients is an indispensable part of formulating surgical treatment plans.

AOPT has been reported to improve health-related quality of life in OLT patients (5). However, fewer studies have been conducted on aspects related to preoperative and postoperative psychological status of OLT patients. The aim of this study was not only to investigate the prevalence of preoperative depression or anxiety symptoms in OLT patients to assess the impact of AOPT on patients’ psychological status, but also to describe the impact of preoperative psychological status on the prognosis, so that it can provide a certain reference to the clinic when formulating treatment plans.

## Patients and methods

### Patients

**Figure 1:**
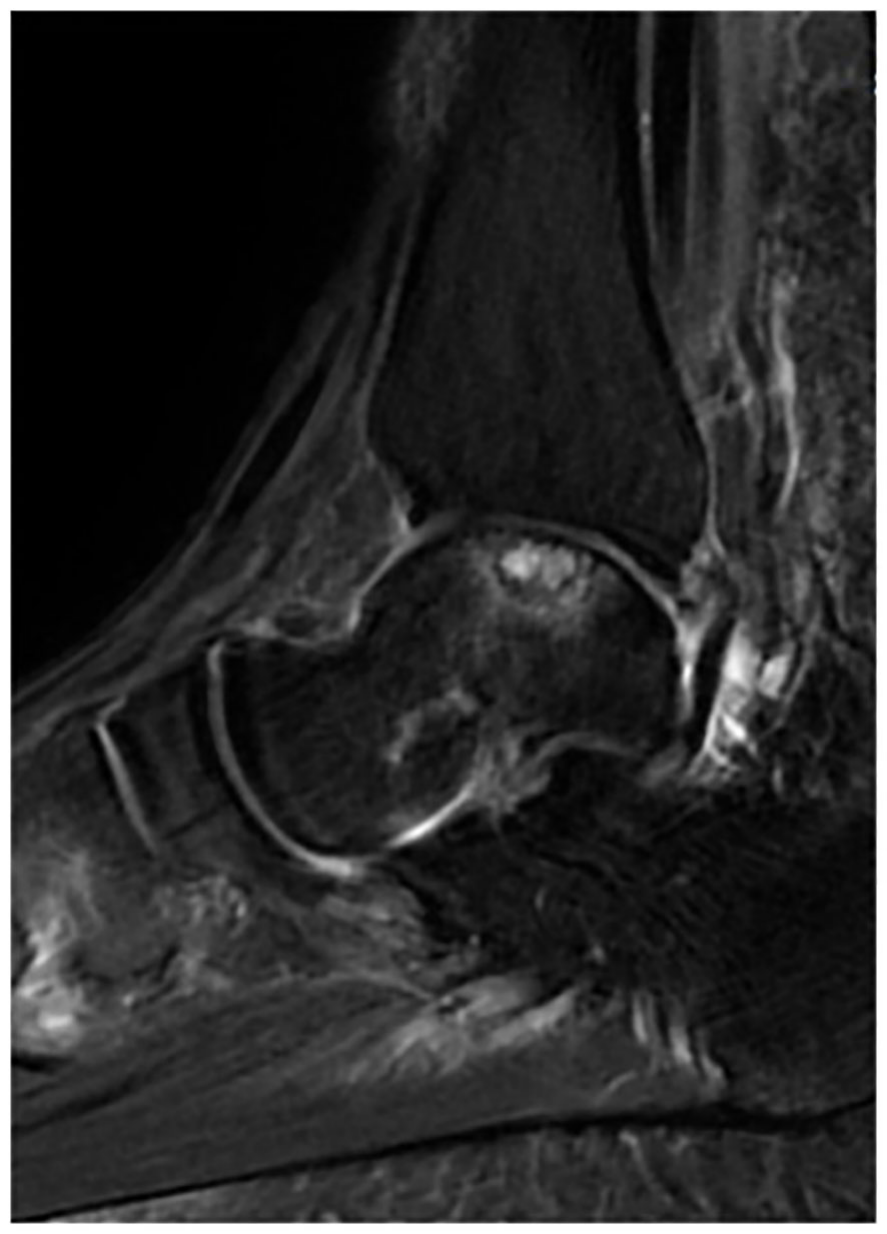
Patient’s preoperative ankle MRI. The patient’s preoperative MRI imaging data, which showed an area of high-density lesion under the talus cartilage, suggested the formation of a subchondral cyst, and the diagnosis of Hepple V OLT was made.

The study was approved by the Ethical Review Committee of Xi’an Honghui Hospital (No:202310008). We retrospectively analyzed patients who underwent autologous periosteal bone grafting in the comprehensive foot and ankle surgery ward of Xi’an Honghui Hospital from November 2021 to May 2023. All patients provided informed consent. The inclusion criteria were as follows: (1) persistent symptoms despite conservative treatment; (2) age >18 years; (3) diagnosis of Hepple V OLT based on imaging data (**Figure 1**); (4) verbal and thinking ability to complete the questionnaire independently; (5) patients with a preoperative American Society of Anaesthesiologist (ASA) grade 1 to 2. The exclusion criteria were as follows: (1) previous history of traumatic surgery on the affected ankle joint; (2) patients suffering from severe osteoporosis, arthritis, and ankle infections; (3) patients suffering from diabetes mellitus, malignant neoplasm, hepatic and renal insufficiency, and other chronic medical diseases; (4) patients who experience traumatic events that affect their psychiatric psyche.

### Methods

We contacted patients via WeChat (Tencent, Shenzhen, China) or telephone and had face-to-face communication with them in order to complete the questionnaire. The American orthopedic foot and ankle society (AOFAS) and pain visual analogue scale (VAS) were used to assess the patients’ pain and overall function preoperatively and at the final follow-up. The AOFAS scoring system is a 100-point system that scores the overall function of the ankle joint through both subjective and objective factors, and is now widely used in the functional assessment of ankle joint disorders before and after surgery. The VAS scoring scale consists of a 100-mm straight line, which allows for an accurate assessment of the patient’s pain level (11). Based on the comparison between the Amsterdam preoperative anxiety and information scale (APAIS) and the Hospital anxiety and depression scale (HADS), we ultimately used the HADS to investigate patients’ anxiety and depression preoperatively and at the final follow-up (12, 13). The HADS consists of two subscales, Anxiety (HADS-A) and Depression (HADS-D), each with 7 entries for a total of 14 entries. Each entry is divided into 4 rating scores (0-3), with a score range of 0-21, and a critical value of 8, with a score greater than or equal to 8 indicating the presence of some anxiety/depression symptoms (12).

For better statistical analysis to understand the impact of psychological status on patient prognosis, we included patients in two separate groups: group A (preoperative presence of anxiety/depression symptoms) and group B (preoperative absence of anxiety/depression symptoms). All patients were anesthetized and operated on by the same group of physicians. Nerve blocks of the sciatic and saphenous nerves were performed using 0.5% ropivacaine. General anesthesia is administered after the nerve block is completed and the block effect is confirmed. Following the surgery, all patients were provided with guidance on specific rehabilitation exercises.

### Statistical analysis

SPSS 25.0 software (IBM, New York, United States) was used to statistically analyze the data. The Shapiro-wilk test was used to assess whether the data conformed to normal distribution. Parametric tests were used to analyze the data if they conformed to a normal distribution, and vice versa, non-parametric tests were used to analyze the data. The independent samples t-test was used to compare the differences in age, duration of illness, and follow-up time between the two groups of patients. A paired-samples t-test was used to assess the difference between preoperative and final follow-up results within the group, and a difference of P < 0.05 was considered statistically significant. The independent samples t-test was used to compare the differences between the preoperative and the evaluation indexes at the last follow-up between the two groups of patients. For patients in the anxiety/depression group, independent samples t-test and Pearson correlation analysis were used to assess the correlation between gender, age, postoperative improvement and psychological status.

## Results

### General condition of the patient

**Figure 2:**
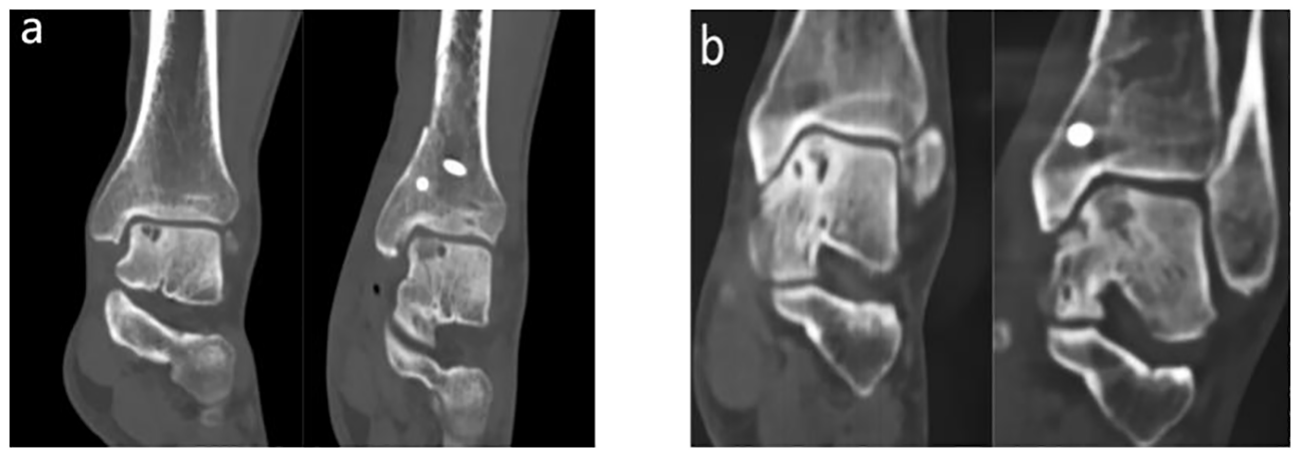
CT imaging data at preoperative and final follow-up. **(A)** CT images of patients in group A at preoperative and final follow-up; **(B)** CT images of patients in group B at preoperative and final follow-up; At the final follow-up in both groups, CT imaging data showed that the periosteal bone and the bone around the talus were well healed.

A total of 57 eligible patients were followed up, with 50 patients completing the full questionnaire survey, including 34 males and 16 females. The average age was 42.50 ± 12.68 years, the average follow-up time was 14.26 ± 4.87 months, and the average duration of illness was 30.10 ± 14.56 months. Anxiety/depression symptoms were present preoperatively in 24 patients (48%), 14 males and 10 females. There were no preoperative symptoms of anxiety/depression in 26 patients (52%), 20 males and 6 females. In terms of donor area selection, all patients were taken from the distal tibia. There were no statistically significant differences between the two groups of patients in terms of age, gender, duration of illness, and follow-up time. The general characteristics of the patients are shown in **Table 1**. Two groups of patients were assessed preoperatively and at their final follow-up visit using CT scans, as shown in **Figure 2**.

**Table 1:** General characteristics of the patients.

	Group A (n=24)	Group B (n=26)	P value
Sex, n(%)			
Male	14(58%)	20(77%)	0.159
Female	10(42%)	6(23%)	
Age (years)	45.67 ± 11.34	39.58 ± 13.36	0.090
follow-up time (months)	14.71 ± 4.90	13.85 ± 4.90	0.537
Duration of illness (months)	30.50 ± 13.90	29.73 ± 15.41	0.854
HADS-A (preop)	9.96 ± 1.43	4.65 ± 1.57	<0.001
HADS-D (preop)	9.71 ± 2.05	4.39 ± 1.96	<0.001

### Analysis of the differences between preoperative and final follow-up assessment indicators

Follow-up was conducted for 7-24 months postoperatively on 50 patients. During the follow-up process, no wound infections, nonunion of bone segments, or nonunion of grafts were observed in either group of patients. In group A, there was a significant improvement in all scores postoperatively compared to preoperative scores (P<0.05, **Table 2**). The HADS-A score decreased from preoperative 9.96 ± 1.43 to postoperative 6.17 ± 1.88, and the HADS-D score decreased from preoperative 9.71 ± 2.05 to postoperative 6.21 ± 2.19. The VAS score decreased from preoperative 64.46 ± 6.32 to postoperative 20.92 ± 6.32. The AOFAS score increased from preoperative 57.08 ± 10.24 to postoperative 85.63 ± 6.83. All of the above comparisons have P < 0.05, see **Table 2**.

**Table 2:** Changes in evaluation indicators of patients in group A before surgery and at the last follow-up.

Evaluation indicators	VAS	AOFAS	HADS-A	HADS-D
preoperative	64.46 ± 6.32	57.08 ± 10.24	9.96 ± 1.43	9.71 ± 2.05
final follow-up	20.92 ± 6.32	85.63 ± 6.83	6.17 ± 1.88	6.21 ± 2.19
t value	28.35	-15.11	10.50	8.97
P value	P<0.05	P<0.05	P<0.05	P<0.05

In group B, the HADS-A score decreased from preoperative 4.65 ± 1.57 to postoperative 3.35 ± 1.70, and the HADS-D score decreased from preoperative 4.39 ± 1.96 to postoperative 2.92 ± 2.04. The VAS score decreased from preoperative 58.65 ± 8.26 to postoperative 14.62 ± 6.12. The AOFAS score increased from preoperative 59.81 ± 10.50 to postoperative 90.04 ± 6.38. In group B, there was a significant improvement in psychological status, pain level, and overall ankle joint function postoperatively compared to preoperative scores (P<0.05, **Table 3**).

**Table 3:** Changes in evaluation indicators of patients in group B before surgery and at the last follow-up.

Evaluation indicators	VAS	AOFAS	HADS-A	HADS-D
preoperative	58.65 ± 8.26	59.81 ± 10.50	4.65 ± 1.57	4.39 ± 1.96
final follow-up	14.62 ± 6.12	90.04 ± 6.38	3.35 ± 1.70	2.92 ± 2.04
t value	36.80	-17.66	9.06	7.24
P value	P<0.05	P<0.05	P<0.05	P<0.05

### Correlation analysis of psychological status

Regarding the correlation between psychological status and prognosis, this study found significant differences in postoperative scores between the two groups of patients (P<0.05, **Table 4**), with patients in group A showing poorer prognosis compared to group B. Patients in group A had lower average VAS scores and AOFAS scores, as well as higher average HADS-A and HADS-D scores, compared to patients in group B. Additionally, we found that patients in group A had higher average VAS scores before surgery compared to group B (P<0.05). However, there was no significant difference between the two groups in terms of average preoperative AOFAS scores (P>0.05, **Table 5**).

**Table 4:** Correlation analysis between psychological state and prognosis.

Evaluation indicators	VAS	AOFAS	HADS-A	HADS-D
t value	3.58	-2.36	5.58	5.50
P value	P<0.05	P<0.05	P<0.05	P<0.05

**Table 5:** Correlation analysis between psychological state and preoperative VAS, AOFAS scores.

Evaluation indicators	VAS	AOFAS
groups t value	-2.773	0.928
P value	P<0.05	P>0.05

**Figure 3:**
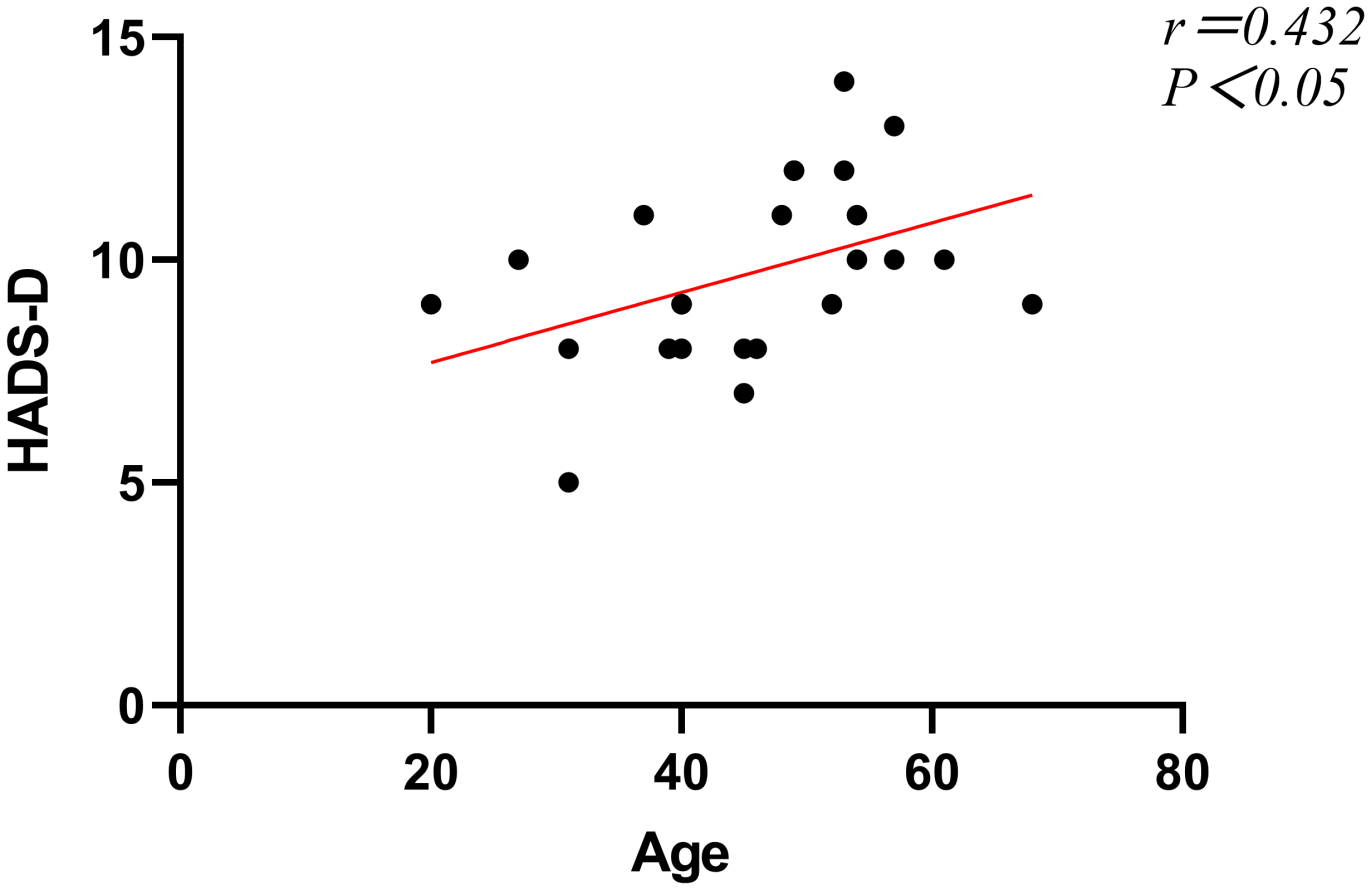
Correlation analysis between age and preoperative depressive symptoms.

For patients in group A, we conducted a study on the correlation between patient gender, age, improvement in various postoperative evaluation indicators, and psychological status. Our study found no correlation between gender and preoperative anxiety levels in patients in group A (P>0.05), but a significant correlation existed between gender and preoperative depression levels (P<0.05, **Table 6**). There was no correlation between age and preoperative anxiety levels (P>0.05), but a significant correlation existed between age and preoperative depression levels (P<0.05, **Table 6**), which showed a positive correlation (**Figure 3**). Furthermore, we discovered that preoperative levels of anxiety and depression did not affect the degree of improvement in postoperative VAS scores, AOFAS scores, and HADS scores (all P>0.05, **Table 7**).

**Table 6:** Correlation analysis between gender, age, and preoperative psychological state in group A patients.

Evaluation indicators	HADS-A	HADS-D
Sex t value	-0.692	-3.034
P value	P>0.05	P<0.05
Age r value	0.106	0.432
P value	P>0.05	P<0.05

**Table 7:** Correlation analysis between postoperative improvement and preoperative psychological state in group A patients.

Evaluation indicators	VAS	AOFAS	HADS-A	HADS-D
Anxiety r value	-0.382	0.028	0.323	0.263
P value	P>0.05	P>0.05	P>0.05	P>0.05
Depressionr value	-0.065	-0.236	-0.293	0.393
P value	P>0.05	P>0.05	P>0.05	P>0.05

## Discussion

OLT often leads to ankle joint pain and may result in disability (14). Especially in Hepple V OLT patients, both the clinical symptoms and the severity of the injuries are more significant. Foot pain significantly impacts the patients’ quality of life, such as limited mobility, impaired balance, and increased risk of falling (15–18). Previous research has reported that patients with foot pain are more likely to experience symptoms of anxiety and depression compared to those without foot pain (15). Moreover, it has been found that there is a certain correlation between psychological conditions and foot pain and function in certain situations (15). For Hepple V OLT, AOPT can be safe and effective in improving patients’ pain and functional activities (19), but few psychological factors are currently taken into account when developing surgical protocols in clinical practice.

Chen et al. found that the mean VAS score after AOPT decreased from 5.40 ± 1.06 to 1.00 ± 1.00 preoperatively (20). Yang et al. reported an improvement in mean AOFAS score from 52.4 ± 12.4 to 90.9 ± 5.2 after AOPT (3). Our study found that both groups of patients showed significant improvement in AOFAS and VAS scores after AOPT. Meanwhile, we also conducted an analysis of the psychological status of AOPT patients. The research results indicated a significant improvement in HADS-A and HADS-D scores after AOPT compared to preoperative scores in both groups. Yang et al. found that no donor site-related complications were observed during the follow-up period (3). We found no complications related to wound infection, non-healing of the osteotomy site and non-healing of the bone graft in the follow-up of both groups. Therefore, we believe that AOPT can be a safe and effective treatment for Hepple V OLT patients.

A total of 50 patients were included in this study, 48 per cent of whom had poor preoperative psychological status. Compared to other chronic diseases (Such as osteoarthritis of the hip, osteoarthritis of the knee, breast cancer, diabetes mellitus) (21–23), patients with Hepple V OLT have a higher incidence of preoperative anxiety/depression symptoms. Harmer et al. study finds significant correlation between preoperative anxiety/depression and prognosis in total hip arthroplasty (23). Kazarian et al. report significant effect of anxiety/depressive symptoms on clinical outcomes after total knee arthroplasty (24). The present study also found a significant correlation between psychological factors and AOPT prognosis. Patients with preoperative symptoms of anxiety/depression had a poorer recovery than patients without preoperative symptoms of anxiety/depression, both in terms of pain, functional activity and psychological status. It has been found that the psychological condition of the patient is related to the level of pain (25). This study demonstrated that although patients’ psychological status did not correlate with comprehensive preoperative functional activity, it did correlate with preoperative pain levels. Therefore, it is necessary to psychologically optimize patients with poor preoperative psychological status in order to help them obtain the best clinical outcome.

Our study of group A patients with poorer psychological status found no significant correlation between age, gender and level of preoperative anxiety. Buonanno et al. found that age does not have a significant impact on patients’ preoperative anxiety, but gender shows a significant correlation with preoperative anxiety levels (26). The reason for some deviation between other studies and the conclusions of this study may be related to the different diseases suffered by the study participants. This study also found a significant correlation between the age, gender, and preoperative depression levels of patients in Group A. Wolf et al. found that long-term physical activity helped reduce the incidence of depressive symptoms (27). Elderly or female patients may have poorer psychological conditions due to relatively less participation in regular physical activities. Studies have reported that the risk of anxiety/depression is twice as high in women compared to men, and female patients tend to experience more severe symptoms with longer durations than male patients (28–30). Furthermore, research has also found that the severity of depression tends to increase with age (31). Although there is a certain correlation between psychological condition and prognosis, the preoperative levels of anxiety and depression do not have an impact on the degree of improvement in various postoperative evaluation indicators. Therefore, it is essential to assess the psychological condition of elderly female patients when encountered in clinical practice, in order to develop personalized treatment plans for these patients.

This study not only investigates the clinical outcomes of AOPT in treating Hepple V OLT but also explores whether there is a correlation between patients’ psychological condition and prognosis. This aims to provide more comprehensive diagnostic and therapeutic services for patients. However, this study has certain limitations. Firstly, this study included only 50 patients with Hepple V OLT, which is a relatively small sample size. Additionally, it is a retrospective study conducted in a tertiary A orthopedic specialty hospital, and the results may not be applicable to patients receiving treatment in other hospitals. To further understand the link between psychological conditions and clinical outcomes after AOPT, we need a large, multicenter, longer-follow-up randomized controlled trial. Secondly, this study only examines the correlation between anxiety/depression and prognosis, and there may be other psychological conditions that have an impact on prognosis.

## Conclusion

AOPT can safely and effectively treat patients with Hepple V OLT. Hepple V OLT tends to cause anxiety/depression symptoms in patients, with higher levels of preoperative depression in female patients than in male patients, and higher levels of depression in older patients than in younger patients, and these psychological factors affect the prognosis of patients. There was no correlation between the degree of preoperative anxiety/depression symptoms and the degree of postoperative improvement.

## Data availability statement

The raw data supporting the conclusions of this article will be made available by the authors, without undue reservation.

## Ethics statement

The studies involving humans were approved by Ethics Committee, Honghui Hospital, Xi’an Jiaotong University. The studies were conducted in accordance with the local legislation and institutional requirements. The participants provided their written informed consent to participate in this study.

## Author contributions

SC: Data curation, Writing – original draft. QZ: Writing – review & editing. JL: Writing – review & editing. YL: Writing – review & editing. BL: Writing – review & editing. HZ: Writing – review & editing. TW: Writing – review & editing. JX: Methodology, Supervision, Writing – review & editing.
